# Assessment of By-Product from *Botryosphaeria rhodina* MAMB-05 as an Effective Biosorbent of Pb(II)

**DOI:** 10.3390/molecules24183306

**Published:** 2019-09-11

**Authors:** Antonio J. Muñoz, Francisco Espínola, Encarnación Ruiz, Aneli M. Barbosa-Dekker, Robert F. H. Dekker, Eulogio Castro

**Affiliations:** 1 Department of Chemical, Environmental and Materials Engineering, Universidad de Jaén, Campus Las Lagunillas, 23071 Jaén, Spain; fespino@ujaen.es (F.E.); eruiz@ujaen.es (E.R.); ecastro@ujaen.es (E.C.); 2 Centre for Advanced Studies in Energy and Environment (CEAEMA), Universidad de Jaén, Campus Las Lagunillas, 23071 Jaén, Spain; 3 Departamento de Química, CCE – Universidade Estadual de Londrina, Londrina – Paraná CEP: 86051-990, Brazil; anelibarbosa@gmail.com; 4 Programa de Pós Graduação em Engenharia Ambiental, Universidade Tecnológica Federal do Paraná, Câmpus Londrina, Londrina – Paraná CEP: 86036-370, Brazil; xylanase@gmail.com

**Keywords:** residual biomass, lead, biosorption, SEM, FT-IR

## Abstract

In this work, two types of biomass preparations (VMSM and M3) from the filamentous fungus *Botryosphaeria rhodina* MAMB-05, which were previously used in a process of production of β-glucan, were assessed as biosorbents of lead. The operating conditions, optimized through response surface methodology and experimental design, were shown to be pH 5.29 and a biosorbent dose of 0.23 g/L for the VMSM biomass type; and pH 5.06 and a dose of biosorbent of 0.60 g/L for the M3 biomass type, at a constant temperature of 27 °C. Fourier transform-infrared spectroscopy analyzed the presence of functional groups on the biomass surface. In addition to give an extra value to the by-product biomass, the VMSM-type from *B. rhodina* MAMB-05 showed an excellent lead biosorption capacity (q_m_) with a value of 403.4 mg/g for the Langmuir model, comparing favorably with literature results, while the M3 subtype biomass showed a value of 96.05 mg/g.

## 1. Introduction

Although major legislative efforts have been made to address the issue, lead and its compounds remain one of the most environmentally problematic heavy metals. Its use in numerous industries causes many natural ecosystems to become contaminated with this toxic metal [1]. The anthropic ecosystems also suffer this problem, generating public health concerns [2]. In 2017 according to the data collected by the Spanish Register of Emissions and Pollutant Sources 25,840 kg of lead were released into the atmosphere and 3,183 kg were released into natural waters [3]. Lead is a metal that can have a powerful toxic effect on living organisms, and also has a bioaccumulative characteristic in that it can be passed onto the food chain [4]. In recent decades, the introduction of process technologies capable of eliminating lead from wastewater has become widespread. Physico-chemical procedures are currently in general use, for example, ion exchange, chemical precipitation, adsorption, oxidation-reduction processes, and membrane technologies among others. These treatment techniques are costly, but are very effective when the contaminant is present in high concentrations [5]. Conversely, lead presents problems when its concentration is low (<100 mg/L), as the effectiveness of its removal becomes very limited [6]. In this case, work on its removal at low concentrations has been carried out since the 1960s on developing ecologically-friendly and low-cost technologies that are also very effective. These technologies can include biosorption of heavy metals [7,8], and have employed several types of biomass (bacteria, fungi, yeasts, algae and plant biomass) that can be active or inactive. When the biomass used is considered a waste product arising from large-scale industrial processes, biosorption takes on an added value [6].

*Botryosphaeria rhodina* MAMB-05, a filamentous, ascomyceteous fungus, is known to produce a variety of bioproducts by submerged fermentation (SmF) when cultivated on different carbon sources [9,10]. This fungal strain was shown to be ligninolytic and could tolerate and degrade high concentrations of aromatic and phenolic compounds, demonstrating its potential for use in the bioremediation of aromatic compounds [11]. In seeking wider applications for this fungal isolate, we were interested in determining whether the mycelial fraction (biomass) could find applications as a biosorbent to remove heavy metals from ecosystems and industrial effluents. This biomass has proven effective in the biosorption of lanthanides [12].

In this work, two types of biomass (VMSM and M3) from *B. rhodina* MAMB-05 grown on different nutrient media containing sucrose were evaluated for their potential in the biosorption of Pb(II) using metal solutions at low concentration of lead (100 mg/L). The two types of fungal biomass were analyzed by Fourier Transform-infrared spectroscopy (FT-IR) to identify potential functional groups that can bind Pb(II). The optimal operating conditions for the biosorption of Pb(II) were determined by Response Surface Methodology (RSM). A kinetic and equilibrium study was also carried out for the biosorption process of Pb(II), and finally, the mechanisms involved in binding lead were identified by scanning electron microscopy (SEM) coupled to an energy dispersive X-ray (EDX) detector.

## 2. Results and Discussion

### 2.1. Fungal Biomass Preparation as Biosorbent

The two fungal mycelial preparations (biomass) of *B. rhodina* MAMB-05 represent the total cellular components, cytoplasm, and cell wall, including residual amounts of fermentation-derived biomolecules from the fungal growth on sucrose as carbon source. The biomass was collected by centrifugation and was not washed, but simply slurried in a small amount of water followed by autoclaving to sterilize the preparation, and then freeze-dried. The difference between the two nutrient media was in the composition of the mineral salts used. VMSM-biomass was produced when the fungus was cultivated on a minimum salts medium developed by Vogel [13]. M3 biomass was obtained using a modified medium [14], which also contained yeast extract (1 g/L). Yeast extract from baker’s or brewer’s yeast is commonly employed in cultivating microorganisms. It is a cell-free extract rich in proteins, amino acids, carbohydrates, vitamins, and minerals. The cell wall of *B. rhodina* MAMB-05 contains a mix of proteins and polysaccharides, among which are α- and β-D-glucans [15]. The different composition of the 2 nutrient media would thus influence the biosorbent properties of the two-biomass preparations, as we demonstrate in this work. 

### 2.2. FT-IR Spectroscopy Study 

FT-IR analysis is based on the excitation of the molecular groups of the biological sample by infra-red (IR) radiation, which causes vibrational movements in molecular bonds: tensions and flexures that can be symmetrical or asymmetrical. The atoms of a chemical bond have vibrational movements that are within the IR range (4000–650 cm^−1^), so that when the frequency of the emitted wave coincides with that of the bond, this radiation is absorbed, generating a characteristic peak in the spectrum that allows us to identify the functional group present in the sample [16]. 

**Figure 1:**
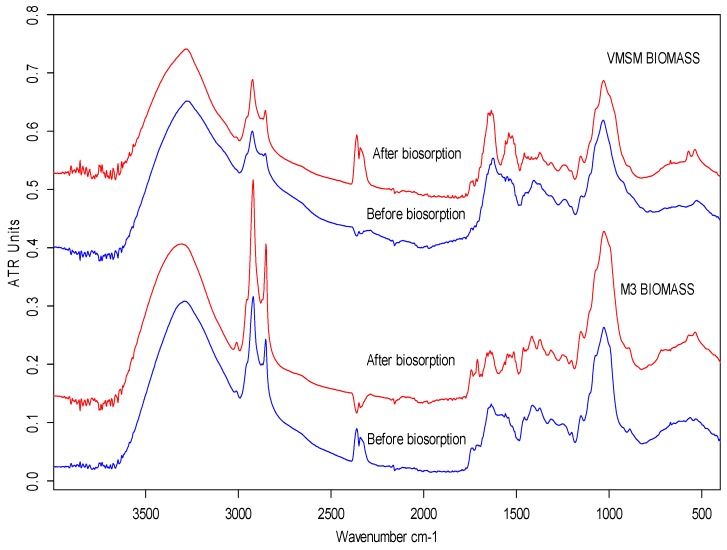
FT-IR spectra of M3 and VMSM biomass preparations derived from *Botryosphaeria rhodina* MAMB-05 before and after biosorption of Pb(II).

Figure 1 shows the FT-IR spectra for biomass preparations M3 and VMSM obtained before and after the biosorption of lead. The spectra reveal a large number of functional groups on the surface of both fungal biosorbents. The functional groups were characterized as amino, carboxylic, carbonyl, hydroxyl, methyl, methylene, and phosphate, and are similar to those reported by others in the scientific literature [17,18,19,20]. The integration of the peaks with the OPUS 7.0 software also showed significant changes in signal strength after biosorption (the data indicating this is not shown). 

The assignment of the main functional groups in the FT-IR spectra for both biomass preparations reveals their involvement in the biosorption, as indicated in Table 1. The main functional groups found were: amino, methyl, carbonyl, and phosphate. Strong involvement of protein bonds C-N, C-O, and N-H (amide I and II), as well as anionic groups such as phosphate and carboxylate were also observed, especially in the VMSM biomass, which appeared to have a greater number of peaks following the biosorption process. Similarly, in the spectral region below 900 cm^−1^, there was a strong shift in some peaks, and new peaks appeared. This region includes the fingerprint region and contains bands that are associated with biological macromolecules, and this presents difficulties in determining what functional groups participate in the biosorption process [21]. However, the involvement of C-H bonds and N-containing bioligands is clearly demonstrated. Deformational modes of CCO groups belonging to carbohydrates may also be involved in biosorption phenomena [22]. 

FT-IR spectral analyses indicated that the two types of fungal biomass presented potential for use in lead bioadsorption. The main changes occurred in the protein region (1200–1800 cm^−1^), which showed the participation of functional groups derived from proteins in the lead biosorption process [20]. However, spectral data from the VMSM biomass preparation implied a greater number of functional groups, and therefore was a priori more effective in the removal of lead from solution. This hypothesis was confirmed in the equilibrium tests.

### 2.3. Optimum Operating Conditions for Pb(II) Bioadsorption: Response Surface Methodology 

An experimental statistical design was carried out to study the optimal operating conditions of the factors involved in the biosorption process. Table 2 shows the experimental results obtained for biosorption capacity (q) in equilibrium conditions ranging from 55.39 to 68.45 mg Pb(II)/g dry biomass for M3 biomass, and 94.93 to 224.91 mg Pb(II)/g dry biomass for VMSM biomass.

From the subsequent statistical adjustment (Design-Expert® software, Minneapolis, MN, USA), Equation (1) for M3 and Equation (2) for VMSM were obtained.

(1) $$q_{e}=-248.235+93.0889\;B+114.531\;pH-10.8924\;B\;pH-32.9522\;B^{2}-10.6679\;pH^{2}$$

(2) $$q_{e}=-832.580+417.015\;B+353.918\;pH-102.359\;B\;pH-49.4909\;B^{2}-28.1801\;pH^{2}$$

where *B* is the biosorbent dose (g/L).

Table 3 shows the results of the analysis of variance (ANOVA) for the M3 and VMSM biomass preparations, and the variability associated with each factor and their interactions. Only statistically significant terms were included; those with *p*-values less than 0.05 (95% confidence level).

The model F-values for both biomass preparations, 201.31 for M3, and 167.13 for VMSM, show that the models were significant, and the lack of Fit F-value, 0.0958 for M3 and 0.1067 for VMSM, show that the Lack of Fit was not significant relative to the pure error. The R-Squared statistic indicated that the models explained 99.41% of the variability for the qe values for M3 biomass, and 99.29% for VMSM biomass. In conclusion, the models appropriately predicted the biosorption capacity as a function of the two considered factors: B (biosorbent dose) and pH. 

**Figure 2:**
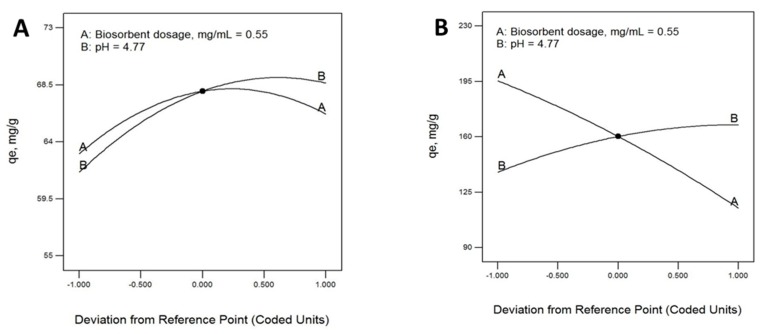
Perturbation plots showing the effect of all investigated factors on lead biosorption capacity by the biomass preparations M3 (**A**) and VMSM (**B**) from *Botryosphaeria rhodina* MAMB-05.

**Figure 3:**
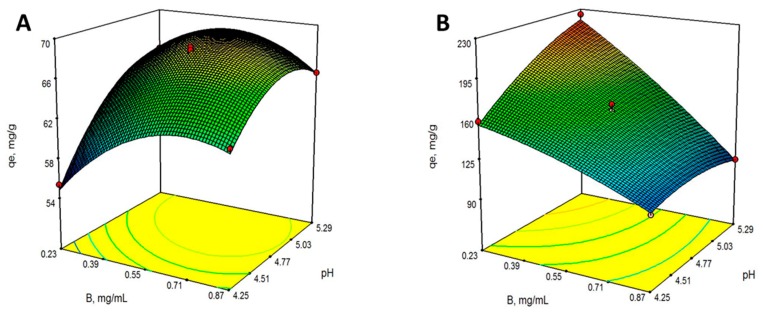
Response surface 3-D plots for Pb(II) biosorption on biomass M3 (**A**) and VMSM (**B**) from *Botryosphaeria rhodina* MAMB-05. The effect of biosorbent dose (**B**), pH, and their reciprocal.

The effects of the factors involved in the biosorption process were also analyzed from *B. rhodina* MAMB-05. The most relevant factor was the dose of biosorbent (B), while the pH of the solution during the biosorption process showed a limited effect within the range studied, especially in the case of the VMSM biomass (Figure 2). This was also shown in the response surface plots for both biomass preparations (Figure 3). A negative effect was observed for the biosorption capacity of Pb(II) with the VMSM biomass preparation as the biosorbent dose was increased.

Beginning with Equations (1) and (2), the optimal operating conditions for biosorption of Pb(II) were obtained for the two types of biomass: pH 5.29 and a biosorbent dose of 0.23 g/L for VMSM; and pH 5.06 and a dose of biosorbent of 0.60 g/L for M3, at a constant temperature of 27 °C. From these values, a respective biosorption capacity of 219.81 and 69.08 mg Pb(II)/g dry biomass was obtained.

### 2.4. Biosorption Kinetics of Pb(II) 

**Figure 4:**
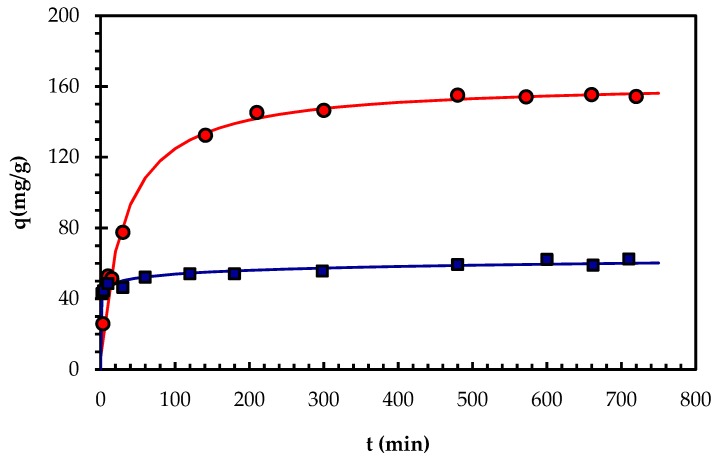
Time profile of Pb(II) biosorption capacity (q) of biomass preparations M3 and VMSM from *Botryosphaeria rhodina* MAMB-05. The symbols (circles: VMSM, squares: M3) are experimental data, whereas the lines correspond to the fitting of the pseudo-second order kinetic for VMSM, and Elovich for M3 models.

The experimental results obtained in the kinetic tests are presented in Figure 4, and show that the biosorption process was fast for both types of biomass preparations. From the nature of the biomass preparations, we deduced that the dominant mechanism would be bioadsorption, and this mechanism markedly depended on the strong contact between the adsorbent and the metal ions of the solution. Despite working under conditions of constant stirring, the characteristics of the biomass did not allow optimum contact until after 5 h. For this reason, the optimum contact time was set at 330 min for all subsequent tests.

The experimental results of the kinetic trials were fitted to three mathematical models as advocated by Vijayaraghvan et al. [21]. Table 5 shows the parameters obtained in the mathematical adjustment considering various boundary conditions [23]). To adjust the experimental data in the mathematical models, linear and non-linear regressions were performed, but the latter offered better adjustments. The results show that for the case of biomass type VMSM, a pseudo-second order is the best model, resulting in a high correlation coefficient for the two integration limits, and with a maximum of 0.994 for the case of: q = q_i_ at t = 0 and q = q at t = t. In the case of biomass type M3, the Elovich model was the best fit with a correlation coefficient of 0.987 for the case: q = 0 at t = 0 and q = q at t = t. The results reported by others [20,22] were consistent with the data obtained in this work. 

### 2.5. Biosorption Isotherm for PBPb(II)

**Figure 5:**
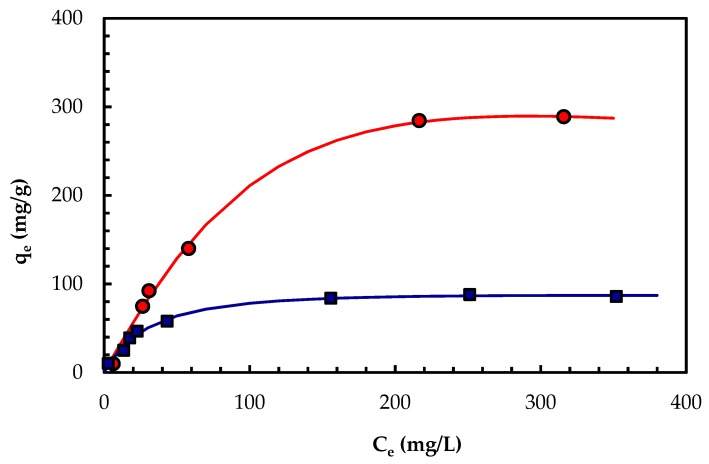
Equilibrium Pb(II) biosorption capacity (qe) of M3 and VMSM biomass preparations from *Botryosphaeria rhodina* MAMB-05 versus different Pb(II) concentrations (Ce). The symbols are experimental data (circles: VMSM, squares: M3), whereas the lines correspond to the Redlich-Peterson isotherms.

Figure 5 shows the experimental data obtained in the equilibrium tests corresponding to the two types of biomass studied. Table 6 includes the parameters obtained by non-linear regression for the different models evaluated. All mathematical adjustments offered high correlation coefficients, but the model that performed best for both types of biomass was that of Redlich-Peterson with correlation coefficients of 0.998 for VMSM, and 0.990 for M3. From the data obtained, it appears that bioadsorption may be taking place in a complex manner involving monolayer adsorption, as well as non-ideal, reversible, and heterogeneous adsorption.

The maximum biosorption capacity according to Langmuir’s model was 403.4 and 96.05 mg Pb(II)/g for the VMSM and M3 biomass preparations, respectively. As shown in Table 4, the results obtained in this work offer maximum biosorption capacities that were above the usual values for other types of microbial biomass biosorbents reported in the scientific literature. The case of the VMSM biomass preparation from *B. rhodina* MAMB-05 is particularly significant, and indicates the potential for its application in the removal of lead, and perhaps other heavy metals too, from contaminated industrial effluents.

### 2.6. Chemical and SEM Analysis of the Biomass Preparations from Botryosphaeria rhodina MAMB-05

**Figure 6:**
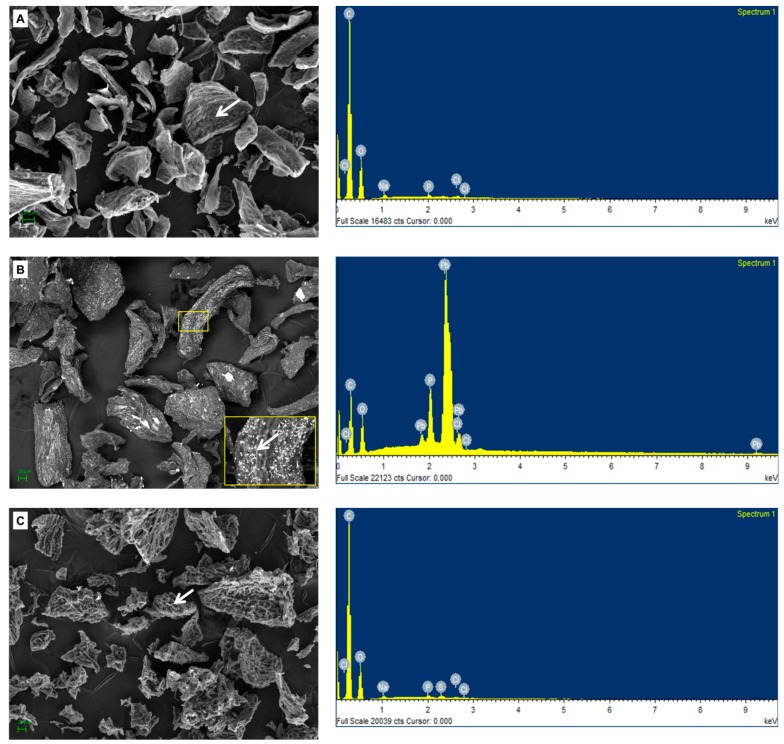

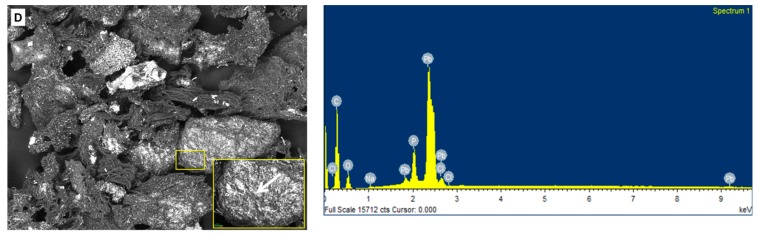
SEM-EDX analysis of M3 and VMSM biomass before (**A**,**C**) and after (**B**,**D**) Pb(II) biosorption. The arrows indicate the area where the EDX spectrum was obtained, and show the presence of lead after biosorption.

The biomass preparations studied here were analyzed by scanning electron microscopy coupled to an EDX detector. Figure 6 show the microphotographs obtained with both biomass preparations. The images show an overview of the fungal biomass samples before (A, C) and after (B, D) the biosorption process, and also the EDX microanalyses obtained in both situations. A large presence of bright particles distributed over the entire surface area can be observed after the biosorption stage with both biomass preparations. In the case of VMSM biomass, these particles were more concentrated on the surface (Figure 6D-inset). Additionally, we can draw another conclusion from the comparison of Figure 6A and Figure 6C that VMSM biomass has a less compact appearance, and this fact may also explain the better results obtained in the biosorption tests with VMSM; having a larger contact surface that would allow better interaction with the lead ions.

The EDX spectra demonstrated that the particles on the surface of the biomass were lead precipitates. The results overall indicate that although the two types of biomass preparations were able to sequester Pb(II) ions, the VMSM biomass was the better biosorbent. The results are consistent with those obtained during experimental trials, and provide graphical evidence that the VMSM biomass showed the best efficacy of the two biosorbents assessed. The data obtained in this work was consistent with the findings presented by others, and demonstrated the potential capacity of this fungal biomass preparation to sequester Pb(II) ions from aqueous solutions (Table 4).

## 3. Materials and Methods 

### 3.1. Production of Fungal Biomass

*Botryosphaeria rhodina* MAMB-05 was maintained on potato-dextrose agar at 4 °C, and sub-cultured at 3-monthly intervals. The fungus was transferred to agar plates containing glucose (10 g/L), minimum salts medium (VMSM) [13] and agar (20 g/L), and left to grow for 4 days at 28 °C. Pre-inoculum was prepared by transferring scrapings of mycelium from the fungal-colonized agar plates to a 250 mL Erlenmeyer flask containing liquid-medium (50 mL) comprising glucose (10 g/L) and VMSM (0.16 mL of a 50× concentrated stock solution), and left to grow at 28 °C under shaking conditions (180 rpm) for 48 h. The mycelium was recovered by centrifugation (1500 × *g*/15 min), homogenized in isotonic saline solution and inoculum prepared as described by Steluti et al. [43]. Aliquots of 10 mL inoculum were used to inoculate each of three 2 L Erlenmeyer flasks containing 800 mL of nutrient medium.

B. *rhodina* MAMB-05 was cultivated in Erlenmeyer flasks by submerged fermentation on two types of liquid nutrient medium: (i) basal medium – VMSM [43] comprising sucrose (50 g/L) and VMSM (20 mL/L), and (ii) M3 comprising sucrose (50 g), NaNO_3_ (3 g), KH_2_PO_4_ (1 g), MgSO_4_.7H_2_O (0.5 g), KCl (0.5), and yeast extract (Oxoid, UK; 1 g) per liter of distilled water [14]. The initial pH of both media was adjusted to 5.8. Fermentation was carried out under shaking conditions (180 rpm) for 72 h at 28 °C.

The fungal cultures were harvested by centrifugation (1500 × *g*/30 min), and the mycelium recovered. Distilled water (100 mL) was added to the fungal biomass and the slurry autoclaved at 121 °C for 20 min. This was followed by lyophilization, and the dried biomass was powdered in a blender, and stored at ambient temperature until required. The 2 types of fungal biomass preparations (designated VMSM and M3) were evaluated for biosorption.

### 3.2. Fungal Biomass Characterization: FT-IR Analysis

In order to identify the presence of functional groups that are potentially involved in the biosorption phenomena, the fungal biomass preparations were analyzed by Fourier Transform Infrared Spectroscopy (FT-IR). Samples of biomass before and after the biosorption process were taken for FT-IR analysis, and subsequently lyophilized and analyzed by attenuated total reflectance (ATR) in a Spectrometer VERTEX 70 (Bruker Corporation, Billerica, Massachusetts, United States), working in the range between 4000 and 400 cm^−1^.

### 3.3. Optimizing Conditions for Biosorption of Pb(II)

The factors involved in the biosorption process were analyzed through a Rotatable Central Composite Design (RCCD) and then analyzed by RSM. The assays were performed using a Pb(II) solution (100 mg/L) under shaking conditions (200 rpm) for 24 h at 27 °C. Factors analyzed were biomass concentration within the range of 0.23–1.00 g/L for each of the 2 types of biomass preparations, and pH ranging from 4.25–5.51. The working solution was prepared with Pb(NO_3_)_2_ in distilled water, and the pH was adjusted with 0.1M NaOH or 0.1M HNO_3_ solutions. The tests were performed in duplicate in 100 mL flasks with a working volume of 50 mL in an orbital shaker (INFORS HT, Ecotron, Infors AG, Bottmingen, Switzerland). After the tests were completed, the samples were filtered through polyethersulfone filters (0.22 µm), and the filtrates analyzed for elemental lead (Pb) by atomic absorption spectrometry (AAS) using an AAnalyst 800 instrument (Perkin Elmer, Midland, ON, Canada). 

The biosorption capacity per unit of dry mass was determined according to Equation (3):

(3) $$q=\frac{\left(Ci-Cf\right)V}{m}$$

where *Ci* is the initial metal concentration; *Cf* is the final metal concentration; *V* is the solution volume (50 mL); and m is the weight of the biosorbent (g, dry basis). 

### 3.4. Biosorption Kinetics

Kinetic tests were carried out to obtain the optimal time of operation in the biosorption process from an initial metallic solution of 100 mg/L. The tests were performed with working volumes of 50 mL of Pb(II) solution in 100 mL flasks at 27 °C and shaken at 200 rpm. The tests were performed in duplicate for each contact time examined over a period ranging from 0 to 720 min. Concentration of dry biomass and pH were selected from the conditions optimized for lead biosorption for each type of biomass preparation used: 0.23 g/L, pH: 5.3 for type VMSM; and 0.60 g/L, pH: 5.0 for type M3. The samples were analyzed as specified in Section 3.3. 

Several kinetic models were considered to adjust the experimental data. The models included pseudo-first order or Lagergren [44], pseudo-second order [23], and Elovich [45]. The kinetic models were tested as outlined in Table 5 using non-linear regression. The software program used was SPSS Statistics version 19 (SPSS Inc., Chicago, IL, USA). When using the models, the adsorption process was assumed to be controlled by chemical reactions and not by diffusion.

### 3.5. Equilibrium Study

The equilibrium tests were performed as described in Section 3.4, but in this case the contact time was set to 330 min, and different lead concentrations were tested that ranged from 10 to 400 mg/L of Pb(II).

Four models: Langmuir [46], Freundlich [47], Sips [48], and Redlich-Peterson [49], were considered (see Table 6) and used to adjust the experimental data. The Langmuir model assumes monolayer adsorption according to a homogenous adsorbent. The Freundlich model is an empirical model that assumes that the adsorbent increases its adsorption surface when the concentration of adsorbent increases, and then it can be applied to multilayers and heterogeneous surfaces. The Sips model takes into account the starting principles of Langmuir and Freundlich and can be used in a wide range of concentrations. Finally, the Redlich-Peterson model is an empirical model that can also be used in a wide range of concentrations, and considers that when β is equal to one, the model obeys the Langmuir isotherm, and when β is equal to zero, Henry’s law is obeyed. The software package SPSS version 19.0 was also used to fit the experimental data.

### 3.6. Field Emission-Scanning Electron Microscopy-Energy Dispersive X-Ray Analysis

In order to understand the topography of the fungal biomass, field emission-scanning electron microscopy (FE-SEM, MERLIN of Carl Zeiss, Göttingen, Germany) coupled to an EDX detector was used. This technique also makes it possible to identify the presence of metal precipitates on the surface of the adsorbent. Biomass samples were taken before and after the biosorption trials, and prepared as described by Muñoz et al. [50].

## 4. Conclusions

The results obtained in this work demonstrate that the reused biomass from the filamentous fungus, *Botryosphaeria rhodina* MAMB-05, is an effective biosorbent of Pb(II). The two types of biomass preparations evaluated gave good results in removing lead ions from aqueous solutions at a concentration of 100 mg/L, with a maximum biosorption capacity according to the Langmuir model of 96.05 mg/g for the M3 type, and 403.4 mg/g for VMSM. The characterization of the biomass and its subsequent analysis by FT-IR identified the involvement of many functional groups in the bioadsorption process. In the VMSM type biomass, the functional groups derived from carboxyl groups (hydroxyl and carbonyl) had a strong implication and could indicate the presence of some type of bidentate chelation with the Pb(II) ions. Likewise, the intervention in the process of bioadsorption of methyl and methylene groups together with groups linked to amides type I and II also seems to be proved. In the M3 biomass there was a lower involvement of functional groups but the participation of methyl and carbonyl groups, and amides type I and II was tested. The FE-SEM-EDX analysis also identified that the VMSM subtype had a less compact appearance and therefore a greater specific surface that could influence a better exposure of the different functional groups.

## Figures and Tables

**Table 1:** The main FT-IR peaks involved in the process of biosorption of Pb(II) by the 2 types of *Botryosphaeria rhodina* MAMB-05 biomass, and possible assignments of the functional groups.

M3 Biomass (peaks cm^−1^)			VMSM Biomass (peaks cm^−1^)				
Before	After	Shift	Before	After	Shift	Functional Groups	Possible Assignment
3290	3303	13				O-H, N-H	Stretching vibrations of amino and hydroxyl groups
			-	3012	3012	-CH_3_	–CH_3_ asymmetric stretching
1792	1786	6	1797	1792	5	C = O	C = O stretching
1771	1778	7				C = O	C = O stretching
			-	1734	1734	C = O	C = O stretching
			1723	1717	6	C = O	C = O stretching
1700	1691	9				-CO. C-N, N-H	-CO, -CN stretching and -NH bending vibration (amide I)
1652	1658	6	1657	1652	5	-CO. C-N, N-H	-CO, -CN stretching and -NH bending vibration (amide I)
1646	1649	3	-	1647	1647	-CO. C-N, N-H	-CO, -CN stretching and -NH bending vibration (amide I)
1636	1642	6	1640	1636	4	-CO. C-N, N-H	-CO, -CN stretching and -NH bending vibration (amide I)
-	1631	1631				-CO	-CO stretching amide I
1559	1564	5	1565	1558	7	N-H, C-N	C–N stretching in amide II group and N–H bending vibration
1542	1547	5	1549	1541	8	N-H, C-N	C–N stretching in amide II group and N–H bending vibration.
1523	1536	13	-	1522	1522	N-H, C-N	C–N stretching in amide II group and N–H bending vibration.
			-	1509	1509	N-H, C-N	C–N stretching in amide II group and N–H bending vibration.
1490	1514	28				-CH	-CH bending
			1484	1480	4	-CH_2_, -CO	-CH bending, symmetric C = O
-	1462	1462	1461	1457	4	-CH_2_, -OH, C = O	-CH bend. Deformations, OH bend. in carboxylic groups, sym. C = O
1457	1453	4				-CH_2_, -CO	-CH bending, symmetric C = O
-	1441	1441	1442	1437	5	-CH_2_, -CO	-CH bending, symmetric C = O
			-	1419	1419	-CH, C-N, C = O	C-H stretching, C-H rocking, C-N stretching
			1405	1397	14	N-H, C-N, COO^−^	N-H bend. in the amine group; C-N stretch.; C = O str (sym) of COO^−^
			1397	1374	5	-CH	C-H bending vibrations
-	1334	1334				-CH	C-H bending vibrations
			1239	1243	4	-PO_2_^−^, -CO	P = O stretching of phosphodiester, or monoester phosphate groups; C-O-C, C-O-P, P-O-P ring vibrations of polysaccharides
			-	1205	1205	C-O-C, C-O-P, P-O-P	C-O-C, C-O-P, P-O-P ring vibrations of polysaccharides
888	893	5				PO_2_^−^, -CH	C-O-C, C-O-P, P-O-P ring vibrations of polysaccharides; C-H bending (aromatic compounds)
-	782	782					Deformational modes of the CCO groups; Nitro compounds and disulfide groups
-	718	718				-CH_2_	C-H rocking
668	673	5	673	668	5		Deformational modes of the CCO groups; Nitro compounds and disulfide groups
			621	617	4		Deformational modes of the CCO groups; Nitro compounds and disulfide groups
			595	603	8		Deformational modes of the CCO groups; Nitro compounds and disulfide groups
			-	571	571	-CH	C-H bending vibrations (aromatics)
562	569	7					Deformational modes of the CCO groups; Nitro compounds and disulfide groups
532	536	4	527	537	10		Deformational modes of the CCO groups; Nitro compounds and disulfide groups
476	467	9	427	420	7		Deformational modes of the CCO groups; Nitro compounds and disulfide groups

**Table 2:** Rotatable Central Composite Design (RCCD) for the optimization of biosorption of Pb(II) by the 2 types of biomass (VMSM and M3) from *Botryosphaeria rhodina* MAMB-05.

Factors		Responses	
B ^1^ (g/L)	pH	q_e_^2^ VMSM Biomass (mg/g)	q_e_ M3 Biomass (mg/g)
0.55	4.77	159.05	67.96
0.55	4.77	159.03	67.53
0.55	4.03	145.76 *	56.69
1.00	4.77	94.93	63.25
0.55	4.77	158.44	67.96
0.55	4.77	159.22	68.45
0.55	5.51	162.95	72.36 *
0.87	4.25	107.59	62.05
0.23	4.25	158.61	55.39
0.10	4.77	202.10	58.70
0.23	5.29	224.91	65.65
0.55	4.77	165.65	68.15
0.87	5.29	105.76	65.06

^1^ B, biosorbent dose; ^2^ q_e_: biosorption capacity; * The strikethrough values were not been taken into account in the statistical adjustment.

**Table 3:** Statistical parameters for the analysis of variance (ANOVA) for the quadratic model of biosorption capacity.

Source of Biomass	Sum of Squares	Degree of Freedom	Mean Square	F-Value	*p*-Value Prob > F
**M3**					
Model	243.16	5	48.63	201.31	<0.0001
B: Biosorbent dose	19.55	1	19.55	80.91	0.0001
pH	59.47	1	59.47	246.19	<0.0001
B pH	13.14	1	13.14	54.40	0.0003
B^2^	73.85	1	73.85	305.72	<0.0001
pH^2^	35.04	1	35.04	145.03	<0.0001
Residual	1.45	6	0.24		
Lack of fit	1.00	2	0.50	4.46	0.0958
Pure error	0.45	4	0.11		
Cor total	244.61	11			
C.V.%	0.77				
R-Squared	0.9941				
Adj. R-Squared	0.9891				
Pred. R-Squared	0.9599				
Adeq. Precision	37.850				
**VMSM**					
Model	155,26.71	5	3105.34	167.13	<0.0001
B: Biosorbent dose	129,38.88	1	129,38.88	696.37	<0.0001
pH	1075.23	1	1075.23	57.87	0.0003
B pH	1160.42	1	1160.42	62.45	0.0002
B^2^	166.59	1	166.59	8.97	0.0242
pH^2^	244.48	1	244.48	13.16	0.0110
Residual	111.48	6	18.58		
Lack of fit	75.06	2	37.53	4.12	0.1067
Pure error	36.42	4	9.11		
Cor total	156,38.19	11			
C.V.%	2.78				
R-Squared	0.9929				
Adj. R-Squared	0.9869				
Pred. R-Squared	0.9211				
Adeq. Precision	41.515				

**Table 4:** Maximum Pb(II) biosorption capacities reported in the scientific literature for different types of biomass preparations.

Biosorbent (Biomass Type)	q_m_ (mg/g)	Reference
*Penicillium* sp. (fungus)	60.77	[24]
*Rhizopus arrhizus* (fungus)	48.79	[25]
*Ceratophyllum demursum* (aquatic plant)	44.80	[26]
Activated carbon-*Enteromorpha prolifera* (green alga)	146.85	[27]
*Bacillus cereus* M^1^_16_ (bacterium)	70.42	[28]
*Aspergillus niger* (fungus)	34.92	[29]
Dried activated sludge	131.60	[30]
*Caulerpa lentillifera* (marine green macroalga)	28.99	[31]
*Cladophora fascicularis* (green alga)	227.70	[32]
*Herbaspirillum. chlorophenolicum* FA1 (bacterium)	151.52	[20]
*Fucus spiralis* (residual biomaterial)	132	[33]
*Bacillus pumilus* (bacterium)	93.24	[34]
*Caulerpa lentillifera* (marine alga)	36.67	[35]
*Mirabilis jalapa* (vegetal leaves)	38.46	[36]
*Escherichia coli* (recombinant bacterium)	108.99	[37]
*Labeo rohita* (fish scales)	196.8	[38]
*Botrytis cinerea* (fungus)	107.10	[39]
*Musa paradisiaca* (vegetal stalks)	13.53	[40]
*Punica geranatum* (vegetal leaves)	18.4	[41]
*Klebsiella* sp. 3S1(bacterium)	140.19	[42]
*Botryosphaeria rhodina* MAMB-05 - VMSM	403.4	this study
*B. rhodina* MAMB-05 - M3	96.05	this study

**Table 5:** Kinetic models tested and kinetic parameters obtained for the biosorption of Pb(II) by the two types of biomass from *Botryosphaeria rhodina* MAMB-05. Integrated equations and boundary conditions considered.

		Parameter	Biomass Preparation	
			VMSM	M3
**Pseudo-first order, or Lagergren** [44]				
	q = 0 at t = 0 and q = q para t = t $q=q_{e}\left(1-e^{-k_{1}t}\right)$	q_e_	149.4	55.059
		k_1_	0.02864	0.6216
		r^2^	0.977	0.899
$\frac{\mathrm{dq}}{\mathrm{dt}}=k_{1}{(q}_{e}-q)$		Σ(q-q_cal_)^2^	847	317
	q = q_i_ at t = 0 and q = q at t = t $q=q_{e}\left(1-e^{-k_{1}t}\right)+\;q_{i}e^{-k_{1}t}$	q_e_	150.4	55.07
		q_i_	12.69	0.5672
		k_1_	0.02211	0.6156
		r^2^	0.984	0.899
		Σ(q-q_cal_)^2^	595	316
**Pseudo-second order** [23]				
	q = 0 at t = 0 and q = q at t = t q=t(1k2qe2+tqe)	q_e_	161.2	56.31
		k_2_	2.246 × 10^−4^	0.02031
		r^2^	0.992	0.934
$\frac{\mathrm{dq}}{\mathrm{dt}}=k_{2}{{(q}_{e}-q)}^{2}$		Σ(q-q_cal_)^2^	295	207
	q = q_i_ at t = 0 and q = q at t = t q=qe−((qe−qi)(1+k2t(qe−qi)))	q_e_	162.5	56.32
		q_i_	6.616	0.2600
		k_2_	2.008 × 10^−4^	0.02026
		r^2^	0.994	0.934
		Σ(q-q_cal_)^2^	229	207
**Elovich** [45]				
	q = 0 at t = 0 and q = q at t = t $q=\frac{1}{b}\ln\left(1+a\;b\;t\right)$	a	15.66	1.026 × 10^6^
		b	0.03659	0.32098
		r^2^	0.982	0.987
$\frac{\mathrm{dq}}{\mathrm{dt}}={a\;e}^{-\mathrm{bq}}$		Σ(q-q_cal_)^2^	666	39
	q = q_i_ at t = 0 and q = q at t = t $q=\frac{1}{b}\ln\left(a\;b\;t+e^{b\;q_{i}}\right)$	a	15.81	1.026 × 10^6^
		b	0.03667	0.3210
		q_i_	−1.210	7.136 × 10^−9^
		r^2^	0.982	0.987
		Σ(q-q_cal_)^2^	665	39

q: biosorption capacity (mg/g) at time t, according to equation 3; q_e_: biosorption capacity (mg/g) at equilibrium; k_1_: pseudo-first-order kinetic rate constant (min^−1^); k_2_: pseudo-second-order kinetic rate constant (g·mg^−1^·min^−1^); a: Elovich constant (mg·g^−1^·min^−1^); b: Elovich constant (g·mg^−1^); r^2^ is the correlation coefficient; Σ(q-q_cal_)^2^ is the sum of the errors squared.

**Table 6:** Isotherm models and biosorption equilibrium parameters for the different isotherm models tested for the biosorption of Pb(II) by the two types of biomass preparations from *Botryosphaeria rhodina* MAMB-05.

Model	Equation	Parameter	Biomass Preparation	
			VMSM	M3
Langmuir [46]	$q_{e}=\frac{q_{m}{\mathrm{bC}}_{e}}{1+{\mathrm{bC}}_{e}}$	q_m_	403.4	96.05
		B	9.252 × 10^−3^	0.03575
		r^2^	0.992	0.988
		Σ(q-q_cal_)^2^	549	75
Freundlich [47]	$q_{e}=K_{F}C_{e}^{1/n}$	K_F_	14.485	15.34
		N	1.874	3.202
		r^2^	0.963	0.915
		Σ(q-q_cal_)^2^	2473	517
Sips [48]	$q_{e}=\frac{K_{s}C_{e}^{1/n}}{{1+a}_{s}C_{e}^{1/n}}$	K_s_	1.219	2.316
		a_s_	3.640 × 10^−3^	2.502 × 10^−2^
		N	0.7541	0.8776
		r^2^	0.996	0.989
		Σ(q-q_cal_)^2^	267	68
Redlich-Peterson [49]	$q_{e}=\frac{K_{\mathrm{RP}}C_{e}^{}}{{1+a}_{\mathrm{RP}}C_{e}^{\beta }}$	K_RP_	2.922	2.910
		a_RP_	3.625 × 10^−4^	1.782 × 10^−2^
		Β	1.513	1.092
		r^2^	0.998	0.990
		Σ(q-q_cal_)^2^	153	60

q_e_: biosorption capacity (mg/g) at equilibrium; q_m_: maximum biosorption capacity (mg/g); b: Langmuir biosorption equilibrium constant (L/mg); C_e_: equilibrium concentrations of metal (mg/L); K_F_: characteristic constant related to the biosorption capacity; n: characteristic constant related to the biosorption intensity; K_s_ and a_s_: Sips isotherm parameters; K_RP_, a_RP_ and β: Redlich-Peterson parameters; r^2^ is the correlation coefficient; Σ(q-q_cal_)^2^ is the sum of the errors squared.
